# Collaborative community mental health and aged care services with peer support to prevent late-life depression: study protocol for a non-randomised controlled trial

**DOI:** 10.1186/s13063-022-06122-1

**Published:** 2022-04-11

**Authors:** Tianyin Liu, Dara Kiu Yi Leung, Shiyu Lu, Wai-Wai Kwok, Lesley Cai Yin Sze, Samson Shu Ki Tse, Siu Man Ng, Paul Wai Ching Wong, Vivian Wei Qun Lou, Jennifer Yee Man Tang, Daniel Fu Keung Wong, Wai Chi Chan, Ricky Yu Kwong Kwok, Terry Yat Sang Lum, Gloria Hoi Yan Wong

**Affiliations:** 1 Department of Social Work and Social Administration, The University of Hong Kong, Pokfulam, Hong Kong, Hong Kong SAR, China; 2 Department of Social and Behavioural Sciences, City University of Hong Kong, 83 Tat Chee Avenue, Hong Kong, Hong Kong SAR, China; 3 Sau Po Centre on Ageing, The University of Hong Kong, 5 Sassoon Road, Hong Kong, Hong Kong SAR, China; 4 Department of Psychiatry, The University of Hong Kong, 102 Pokfulam Road, Hong Kong, Hong Kong SAR, China; 5 Hong Kong Metropolitan University, 30 Good Shepherd Street, Hong Kong, Hong Kong SAR, China

**Keywords:** Older adults, Depressive symptoms, Collaborative stepped care, Peer support, Prevention, Early intervention, Effectiveness, Cost-effectiveness

## Abstract

**Background:**

Late-life depression is common, modifiable, yet under-treated. Service silos and human resources shortage contribute to insufficient prevention and intervention. We describe an implementation research protocol of collaborative stepped care and peer support model that integrates community mental health and aged care services to address service fragmentation, using productive ageing and recovery principles to involve older people as peer supporters to address human resource issue.

**Methods/design:**

This is a non-randomised controlled trial examining the effectiveness and cost-effectiveness of the “Jockey Club Holistic Support Project for Elderly Mental Wellness” (JC JoyAge) model versus care as usual (CAU) in community aged care and community mental health service units in 12 months. Older people aged 60 years and over with mild to moderate depressive symptoms or risk factors for developing depression will be included. JoyAge service users will receive group-based activities and psychoeducation, low-intensity psychotherapy, or high-intensity psychotherapy according to the stepped care protocol in addition to usual community mental health or aged care, with support from an older peer supporter. The primary clinical outcome, depressive symptoms, and secondary outcomes, self-harm risk, anxiety symptoms, and loneliness, will be measured with the Patient Health Questionnaire-9 (PHQ-9), Self-Harm Inventory, Generalized Anxiety Disorder 7-item scale (GAD-7), and UCLA Loneliness 3-item scale (UCLA-3) respectively. Cost-effectiveness analysis will assess health-related quality of life using the EQ-5D-5L and service utilisation using the Client Service Receipt Inventory (CSRI). We use multilevel linear mixed models to compare outcomes change between groups and calculate the incremental cost-effectiveness ratio in terms of quality-adjusted life years.

**Discussion:**

This study will provide evidence about outcomes for older persons with mental health needs receiving collaborative stepped care service without silos and with trained young-old volunteers to support engagement, treatment, and transitions. Cost-effectiveness findings from this study will inform resource allocation in this under-treated population.

**Trial registration:**

ClinicalTrials.gov NCT03593889. Registered on 20 July 2018.

**Supplementary Information:**

The online version contains supplementary material available at 10.1186/s13063-022-06122-1.

## Background

Depression is one of the most common yet under-recognised chronic conditions among older people. In Europe, for example, a meta-analysis found an overall prevalence of 12% of depression among older people [1]. In the East, for instance, in Hong Kong, approximately 1 in 10 older people has clinically significant depressive symptoms [2]. Depression is prevalent in older people due to increased vulnerability in later life, including multiple risk factors such as loneliness, lack of social interaction, lack of enjoyable/meaningful activities, chronic pain, chronic diseases, and bereavement [3]. With population ageing, the number of older people is projected to double to 1.5 billion in 2050 globally [4]; if no action is taken, the number of older persons with depression could increase, causing impairments and reduced quality of life for the individuals concerned and their caregivers, and impose a heavy burden on care systems.

Prevention and early intervention are effective for late-life depression and can reduce suffering and societal costs [5]. If untreated, even mild depressive symptoms can lead to an 81% increase in suicidal risks [2], and late treatment cannot avert two-thirds of the disease burden [6]. In the US, the increased prevalence of major depressive disorder (MDD) in people aged over 50 years in five years has resulted in a 62% increase in direct cost, or a US$44,411 million increase, without taking into account the additional or indirect cost amounting to 6.6 times the direct cost [7]. Earlier models suggested that it is theoretically possible to prevent one older person from developing MDD for every 5.3 older people at high risk receiving “selective prevention” measures and for every 3.2 older people with mild symptoms receiving “indicated prevention” measures [6]. Empirical evidence with stepped care interventions suggests that the incidence of MDD can reduce by as much as 50% compared with care as usual [8].

Despite the promise of prevention and early intervention for old age depression, there are multiple challenges to its implementation as a routine service. First, system fragmentation is a common barrier in mental health care in older age, as multiple service sectors and providers are often involved [9]. For example, in Hong Kong, in theory, older adults with mental health needs can receive specialist mental health and psychiatric services, community mental health services, and/or community elderly services, all provided by different units [10]. However, in reality, there are no clear triage systems or protocols to assess the severity level for making referrals. As a result, older people with varying degrees of depressive symptoms queue for or receive services of similar intensity, causing service overload and deterioration in clients’ depressive symptoms during the lengthy waiting times [11]. Second, the gap between geriatric mental health needs and a professionally trained workforce providing adequate and appropriate services is growing [12]. For instance, a 2010 survey in the USA revealed a shortage of 45,000 psychiatrists [13]. In Hong Kong, Chan [11] reported a ratio of only 4.39 psychiatrists and 29.15 mental health nurses per 100 000 population. Third, older adults with depression may face double jeopardy in a culture that stigmatises mental illness and advanced age, which impedes help-seeking [14]. Research shows that older adults are less likely than younger people to self-identify mental health problems or seek mental health services [15]. In an Asian society such as Hong Kong, this problem is further compounded by the traditional “face-saving” culture, where seeking professional mental health help is often associated with “losing face” [16], and most older adults often choose to maintain secrecy regarding their condition, instead of seeking help [17].

### Collaborative stepped care, task shifting, and productive ageing for preventing depression

These challenges can theoretically be addressed using concepts of collaborative stepped care, task shifting, and productive ageing.

Collaborative care refers to a systematic approach that integrates different mental health professionals and/or other care extenders that has been shown to be effective in improving outcomes of patients with depression [18, 19], including older adults [20, 21]. However, there is noticeable heterogeneity in the service units to be integrated and forms of collaboration. For example, the Improving Mood–Promoting Access to Collaborative Treatment (IMPACT) collaborative care management programme for late-life depression in the USA introduced the role of depression clinical specialist, who coordinated care provision, supported the primary care provider, and acted as a bridge between the primary care and psychiatric teams [22]. In the UK, a similar approach to IMPACT was adopted by engaging a community psychiatric nurse as a care coordinator, who liaised with the GP and coordinated self-help intervention [20]. Stepped care is an approach that takes into consideration both clinical outcomes and efficiency in resource allocation [23], and it provides service users with interventions ranging from the least to the most resource-intensive, based on the severity and complexity of their health conditions [24]. With its emphasis on matching the intensity of intervention to service users’ level of need, the stepped care approach illustrates the potential to prevent the development and progression of sub-threshold depression in older people through the provision of interventions along a continuum of care with varying degrees of intensity [25]. A notable initiative in this respect is the Improving Access to Psychological Therapies (IAPT) programme launched in 2008 in England that has largely increased service access [26]. In 2017, it treated over 560,000 patients per year and obtained clinical outcome data on 98% of these individuals, of whom 50% showed recovery, and two-thirds showed worthwhile benefits [27, 28].

Stepped care such as IAPT also allows for task shifting, a possible solution to the challenge of the shortage in the professional workforce [29]. Task shifting includes shifting service delivery of specific tasks from professionals with higher qualifications to less-well qualified personnel or creating a new group of workers with specific training [30]. The involvement of service users or people with lived experience of psychiatric illness supporting others in their recovery from mental health problems, such as peer support workers, also enhances service capacity [31]. Reviews of literature suggest that peer support workers can reduce hospital admissions among those with whom they work [32]. To identify and support other older people at risk of or with depression, peer supporters may also be better positioned to reach out to and engage “hidden” older adults in Chinese societies where stigma surrounding mental health problems is high.

We have designed a collaborative stepped care and peer support mental health service model for the identification, prevention, and early intervention of depression in older people in Hong Kong to synthesise this theoretical and empirical knowledge. In contrast to existing collaborative stepped care models, this new service focuses on prevention and early intervention, and hence the integration of services takes place at the community level, with collaboration between two types of service (i.e. aged care services and community mental health services) operated by different organisations. The new service also has a peer supporter programme based on the principles of task shifting and productive ageing. We aim to investigate the effectiveness and cost-effectiveness of this new service in an implementation study (Jockey Club Holistic Support Project for Elderly Mental Wellness, JC JoyAge).

## Description of the JC JoyAge Intervention

### Service model development: collaborative stepped care and peer support model

**Fig. 1:**
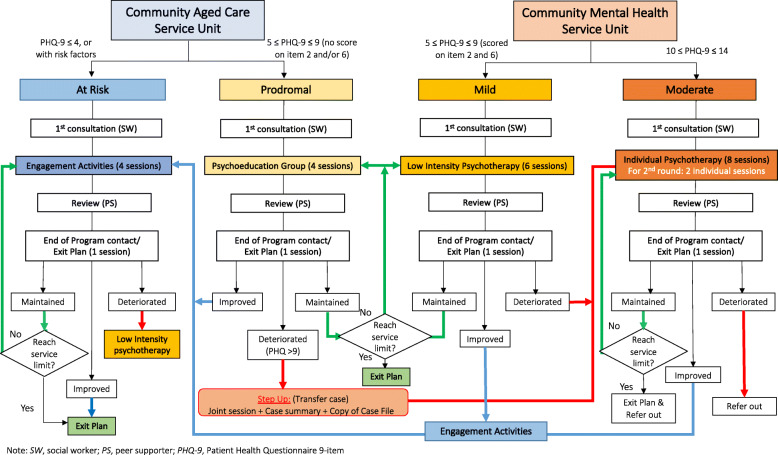
Collaborative stepped care and peer support services flowchart

We developed the collaborative stepped care model through a co-creation process involving multidisciplinary researchers and service providers (frontline and management) from community aged care and mental health care services. In the finalised model, preventive intervention services will be provided by trained social workers and peer supporters from aged care or mental health service units according to level of risks, symptom severity, and intervention response. A community mental health service unit and a community aged care service unit will collaborate to deliver the service for step-up and step-down cases. Fig. 1 summarises the stepped care model in JC JoyAge. We have developed a clinical protocol and guidelines for coordinated care among community mental health and aged care services for elderly mental wellness, based on ageing and mental health theories and the grounded feedback and practice wisdom from the social workers.

### Capacity building and workforce development

#### Training for social workers

A 256-h theoretical and clinical training course is developed to equip project social workers with the competence to provide mental health services to older people. The training course consists of both theoretical sessions and practical sessions. The theoretical sessions involve three modules: (1) societal issues and public mental health approach to late-life depression, (2) current knowledge on mental health in old age, and (3) assessment, case identification, and intervention in late-life depression. Following the theoretical sessions, weekly practical sessions cover (1) use of individual screening and assessment tools; (2) case-based learning of systematic case intake and formulation in older adults and eliciting possible hidden psychopathology; (3) supervised clinical practice, demonstration, and role play for clinical intervention; (4) regular case conferences for care planning; and (5) supervised clinical practice and role play for engaging older people at risk of or with depression.

#### Training for peer supporters

Peer supporters are recruited through non-governmental organisations (NGOs) in the community. The inclusion criteria for peer supporters are (1) aged 50 years or older; (2) able to read and write Chinese and fluent in spoken Cantonese; (3) willing to share their experience and have good basic communication skills; and (4) have some knowledge about the local district. A 100-h training course is developed to equip the peer supporters with sufficient knowledge and skills to collaborate with social workers in delivering the services. The training consists of two phases. Basic training focuses on theoretical knowledge, e.g., old age depression, peer support, productive ageing, coping with adversity and life events in old age, and help-seeking. Advanced training focuses on practical knowledge, e.g., motivational interviewing and focusing therapy. Peer supporters receive concurrent didactic lectures and practicum sessions (e.g. street booths, telephone calls, and home visits) in both training phases. The trained peer supporters will provide outreach and engagement services at the service level, offering assistance and emotional support to older people at risk of or with depression. They will increase service units’ capacity at the system level by taking up the semi-skilled and labour-intensive work, enabling social service staff to focus on professional and management level work in elderly mental wellness. At the community level, peer supporters will positively influence their family and social networks in the neighbourhood to enhance mental health literacy [33].

### Outreach and engagement activities in the community

Based on local experience in detecting and engaging older people at risk of depression, effective outreach programmes will be delivered in the format of general health screening in the neighbourhood area, and events on topics of concern to at-risk older adults (e.g., chronic pain, sleep). Trained peer supporters will undertake home visits to detect and engage hidden cases. This will be in the format of generic health promotion or other pragmatic approaches to lower mental health service stigma and barriers to help-seeking. Depression risk assessments will be provided as part of these activities to detect these otherwise hard-to-reach, hard-to-engage older adults.

### Screening, intake assessment, and triage

Participants will be recruited through community outreach and open referral. Those who meet the eligibility criteria will be asked to give informed consent to participate in the study. Upon doing so, they will undergo a clinical intake and assessment interview conducted by a clinical social worker. The assessment battery will include instruments that measure depression, suicidal risks, and other outcomes (please refer to the programme evaluation section for more details). The initial intervention provided for participants will be determined by their depression level indicated by their scores on the Patient Health Questionnaire (PHQ-9) [34] during the intake assessment.

### Selective prevention, indicated prevention, treatment, and referral

Trained social workers and peer supporters from aged cared or mental health service units will provide collaborative stepped care to clients according to their level of risks, symptom severity, and intervention response. According to the project clinical protocol, social workers will perform professional needs and risks assessment to ensure triage and programme selection. Table 1 and Fig. 1 outline the collaborative stepped care workflow. Adherence to the service model is monitored through monthly case meetings, on-demand clinical supervision, and an electronic data input system. Any deviation from the service model, e.g., unmatched service to the client’s intake scores, will be flagged for the supervisor’s review and approval before processing the client’s data.

**Table 1:** Service care model and collaboration between service units

Client group	Criteria	Intervention	Service unit(s)	Service period
At Risk	PHQ9 ≤ 4 or with risk factors	Selective prevention: engagement activity + PS	Community aged care	2–9 months
Prodromal	5 ≤ PHQ ≤ 9 without score on item 2 or 6	Indicated prevention: psycho education + PS	Community aged care	3–9 months
Mild	5 ≤ PHQ ≤ 9 and scored on item 2 and 6	Indicated prevention: group psychotherapy + PS	Community mental health care	3–9 months
Moderate	10 ≤ PHQ ≤ 14	Individual psychotherapy + PS	Community mental health care	6–12 months
Moderately severe and above*	PHQ ≥ 154	Care as usual	Traditional mental health service, HA	N. A

*Beyond the scope of the current project, mainly referral for appropriate services

*PHQ* Patient Health Questionnaire, *PS* Peer Supporter, *HA* Hospital Authority

#### Selective prevention (2–9 months)

Older people with milder conditions (“vulnerable” state), operationalised as a PHQ-9 score of 4 or below, and having one or more risk factors of old age depression, will receive a 4-week “selective prevention” group (group size: 6–8), a mental health information package after completing the group, and a review at week 6–8. Peer supporters will lead the group with social worker supervision at the aged care service unit, and the group content covers wellness topics tailored to the person’s concern (e.g. coping with insomnia, exercising). Participants will be discharged at week 8 if they exhibit improvements in depressive symptoms or have reduced risk. If they show no change, they will enrol in another 4-week group, followed by a review until they reach the service limit (9 months). If participants show increased depressive symptoms at review, they will be stepped up to receive indicated prevention.

#### Indicated prevention (3–9 months)

Older people at the prodromal or mild stage of depression will receive structured low-intensity psychoeducation followed by a review at aged care service units, delivered by trained social workers and supported by peer supporters. Older persons who are at the prodromal stage, operationalised as having a PHQ-9 score of 5 to 9 but do not have negative mood or thoughts (as indicated by items 2 and 6 of the PHQ-9, respectively) will receive a 4-session psychoeducation group, with an approximate group size of 10 people, on one of the following topics (pain, sleep, stress, physical activities, and bereavement) based on their needs. Older persons at the mild stage of depression, operationalised as having a PHQ-9 score of 5 to 9 with the presence of negative mood and thoughts, will receive 6-session low-intensity psychotherapy such as cognitive behavioural therapy (CBT), problem-solving therapy (PST), and reminiscence therapy in groups of 6 to 8 persons. After the review at weeks 10 to 12, participants will be stepped down to receive selective prevention if they exhibit improvements in depressive symptoms. If they show no change, they will enrol in another round of indicated prevention, followed by a review until they reach service limits (9 months). If participants show increased depressive symptoms at review, they will be stepped up to receive treatment.

#### Treatment and referral (6–12 months)

Older persons with moderate depression, operationalised as having a PHQ-9 score of 10 to 14, will receive 8 sessions of CBT, either in individual or group format, provided by the trained social workers at mental health care units, followed by a review. After the review at weeks 10 to 12, participants will be stepped down to receive selective or indicated prevention if they exhibit improvements in depressive symptoms. If they show no change, they will enrol in another round of treatment, followed by a review until they reach service limits (12 months). If participants show increased depressive symptoms at review, they will be referred to existing services. Peer supporters will be matched to individual older adults to walk them through the process and provide regular follow-up for 1 year maximum. Older persons who have a PHQ-9 score of 15 or above will be referred to existing specialist mental health services in the community. Those with suicidal risks will be referred to a hospital, mainly through the Fast Track Clinic for Elderly Suicide Prevention Programme.

### Objectives

This study aims to (1) examine the effectiveness of the collaborative stepped care and peer support model in improving depressive symptoms, self-harm risk, anxiety symptoms, and loneliness and (2) evaluate the cost-effectiveness of the collaborative stepped care and peer support model in terms of quality-adjusted life years as compared with care as usual (CAU) among community-dwelling older adults aged 60 years and over at risk of or with depressive symptoms.

### Methods

#### Study design and procedures

We use a non-randomised controlled trial to compare older adults at risk of or with sub-threshold symptoms receiving JoyAge service (intervention group) with those receiving CAU (control group). The intervention study will be conducted in four districts (out of 18) in Hong Kong. Participants residing in the four piloting districts will be recruited through six partner NGOs to the intervention group by social workers, and members of the same NGOs residing in other districts will be recruited as control subjects by researchers to increase the compatibility of the two groups. No randomisation is applied due to the clinical nature of the study and several feasibility considerations, for example, a new collaborative workflow between different service units, workforce training, and the high possibility of intervention and control group participants to interact outside treatment groups. Data collection will take place at three time points: baseline, discharge (applicable to the intervention group only), and 12-month follow-up from baseline. Since the JoyAge service is stepped care with peer support, the length of the service will depend on individual client’s progress, and it could vary from 2 to 12 months. To promote participant retention, we will inform participants from both groups about the approximate date of the follow-up assessment. We will also send out postcards to the CAU group in special holidays, e.g., Chinese Lunar New Year, to remind them about the study since they do not receive service from the JoyAge project. Ideally, a follow-up interview will be conducted face-to-face at the client’s convenience and preferred venue; however, a telephone interview will also be an option if the client prefers so. Each client will be contacted initially by phone to arrange a follow-up interview, and a reminder call will be made 1 day prior to the interview. If a client cannot be reached by phone after three calls, social workers of the NGO where the client is recruited will step in and check the client’s status via home visits or other means. For an overview of the schedule of enrolment, interventions, and assessments, see Table 2.

**Table 2:**
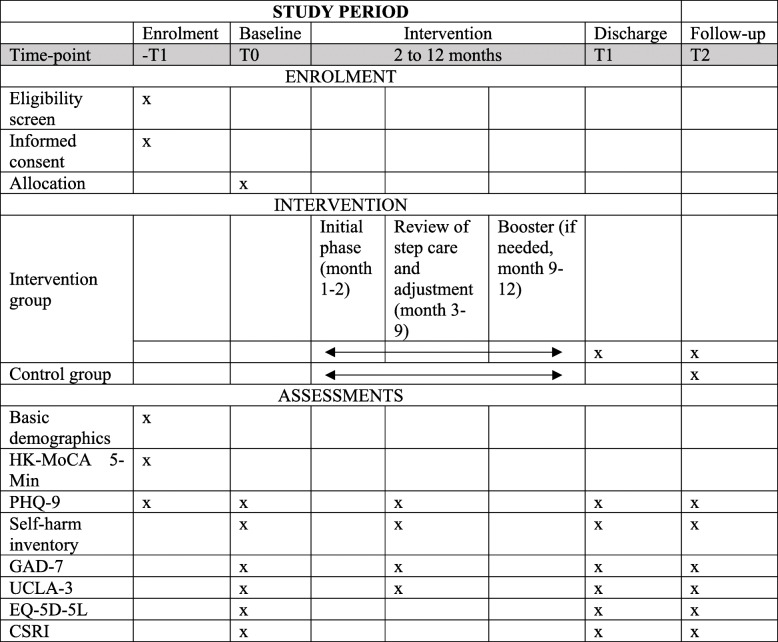
SPIRIT schedule of enrolment, intervention, and assessments of JoyAge trial

*HK-MoCA 5-Min*, Hong Kong Montreal Cognitive Assessment 5-Minute Protocol; *PHQ-9*, 9-item Patient Health Questionnaire; *GAD-7*, Generalized Anxiety Disorder-7; *UCLA-3*, 3-item University of California, Los Angeles (UCLA) loneliness scale; *EQ-5D-5L*, European Quality of Life five-dimensional questionnaire, five-level version; *CSRI*, Client Service Receipt Inventory

### Ethical considerations

This study has been approved by the Human Research Ethics Committee (HREC) of the University of Hong Kong (Reference number: EA1709021). The study is registered with the US National Institute of Health (NIH) ClinicalTrials.gov (NCT03593889) on 20 July 2018. The trial protocol fulfils the Standard Protocol Items: Recommendations for Interventional Trials (SPIRIT) guideline [35] (Supplementary Table 1) and World Health Organization Trial Registration Data Set (Supplementary Table 2). Social workers and peer supporters will seek written consent from the participants before they join the trial. If any changes are made to the protocol, we will make an amendment both to the HREC of the University of Hong Kong and on the ClinicalTrials.gov. Frontline service providers, i.e. social workers and peer supporters, will contact the participants and solicit their informed consent anew.

There are no greater anticipated negative effects in psychological interventions in JoyAge services, such as strong but temporary feelings of distress and deterioration, than care as usual [36, 37]; and there are no known hazards or discomforts associated with the intake, discharge, or 12-month follow-up assessments. All assessments will be conducted by trained social workers or research assistants. Training will be provided for them before data collection, and detailed study management protocols developed by the research team independent of the sponsors will be used to monitor data. Hardcopies of clients’ data will be stored in locked rooms in NGO centres and the university in cabinets with lockers, with access restricted to the responsible workers and research team. All data will be uploaded to a secure server and access to the final dataset is restricted to the research team. All computers used in this project will be password protected, and all data collected will be anonymised for analysis purposes. There will be a database backup weekly and stored in a different secure server at the University of Hong Kong. A double data entry mechanism is developed, 10% of the data will be randomly selected and checked via double entry, if the error rate is over 0.5%, research assistants will examine the hard copies and re-enter the data.

**Fig. 2:**
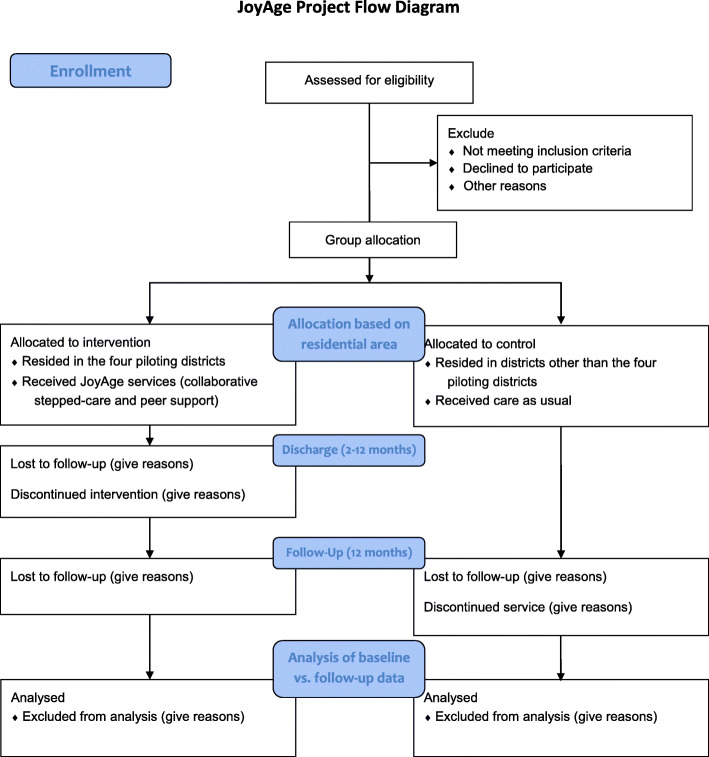
Consolidated Standards of Reporting Trials (CONSORT) flow chart of JoyAge trial

During the course of this study, a participant who does not respond to intervention positively after nine months will be referred to existing services. Participants may withdraw from the study at any time point. Social workers and peer supporters will regularly follow up with the clients and perform risk assessment; the frequency may vary between weekly and monthly depending on the clients’ needs and social workers’ clinical judgement. If at any time during the study, there are concerns about a person’s suicidal risk, or if the person is assessed as having moderate or higher risk of suicide, referral will be made to a hospital or Fast Track Clinic for Elderly Suicide Prevention Programme [38]. Project social workers will also follow their organisation’s protocol for risk management and inform their service supervisor or responsible staff member. These cases will be considered as drop-outs of the study, and corresponding social workers will mark down their drop-out reasons and adverse events, if any, in the exit form. All adverse events will be reported in the CONSORT flow chart (Fig. 2) as reasons to exclude the participants from the analyses after they are recruited to the study. After completion of the trial, all participants will become service users or members of the partner NGOs, they will receive regular events updates from the NGOs and receive continued care or referral as needed in accordance to the public service provision requirement.

### Study setting

The intervention study will be conducted in community setting in four districts (out of 18) in Hong Kong. In each district, a community mental health service unit and a community aged cared service unit will collaborate to provide a coordinated service to older adults at risk of (the prevention group) or having mild-to-moderate level of depressive symptoms (the treatment group). All service units are NGOs, and there are six NGOs participating in this study (listed in ClinicalTrial.gov as collaborators). The prevention group, comprising adults aged 60 years or older who are in a vulnerable state (exposed to increased risk factors, such as recent spousal death, disability, chronic illness) or who have sub-syndromal depressive symptoms plus risk factors, such as disability, chronic illness or living alone, will receive preventive intervention at community aged care centres. The treatment group will comprise two subgroups: those with mild to moderate sub-threshold depressive symptoms without immediate risks who will receive care at aged care service centres and those with moderate-to-severe sub-threshold depressive symptoms who will receive care at the community mental health service centre. Older adults in this programme will be able to move up or down the stepped care levels and between the different types of service according to needs and risks. They will receive assertive preventive care and treatment with a clear goal for recovery. Across levels of care and services, peer supporters will establish rapport, guide and support older adults in the programme and offer longer-term engagement (up to one year within the programme including follow-up).

Participants in the control group will be recruited from the community aged care units and mental health service units of the six partner NGOs in districts other than the four piloting districts of Hong Kong, and they will receive care as usual (CAU). CAU in community aged care units includes health education, case management, social and recreational activities, and other tangible support like meals and laundry services etc. CAU in community mental health care units includes casework counselling, clinical psychological services, and referral to the community psychiatric services of the Hospital Authority for clinical assessment or psychiatric treatment. These services are all district-based, and there is a limited collaboration between the two service units. Continuation and provision of the intervention model after the trial is subject to funding opportunity. If the government adopts the JoyAge model as part of the regular service, it will be available to all eligible older adults in Hong Kong; if no additional funding can be secured, there is no plan for post-trial care.

### Participants

The inclusion criteria for participants are (a) aged 60 years or older; (b) at risk of depression, or have sub-threshold depressive symptoms based on assessment by social workers; and (c) able to give informed consent to participate. The exclusion criteria are (a) known history of autism, intellectual disability, schizophrenia-spectrum disorder, bipolar disorder, Parkinson’s disease, or dementia/significant cognitive impairment; (b) (temporary exclusion criteria) imminent suicidal risk; and (c) difficulty in communication.

### Sample size

Based on previous work in stepped care for depression, we separate the sample size estimation for preventive care and intervention groups. For preventive care, meta-analyses of 47 depression prevention trials revealed an effect size of Pearson’s *r*=0.15 from pre-to-post and *r*=0.11 from pre-to-follow-up comparisons [39] (equivalent to Cohen’s *d*=0.30 and 0.22, respectively); for treatment, meta-analyses of 10 studies revealed a moderate effect size of Cohen’s *d*=0.34 (95% CI 0.20–0.48) with pooled 6-month between-group effect [40]. We set the allocation ratio as 4:1 (intervention to control) based on principles to maximise trial participant’s benefit [41] and operational considerations. Assuming a small effect size of 0.2 for preventive care, and a moderate effect size of 0.3 for treatment, with *α* = 0.05, power of 0.80, and allocation ratio of 4:1, 246 participants are needed for control and 984 for intervention for preventive care category, and 110 as control and 440 as an intervention for the treatment category. Assuming a total non-consent and dropout rate of around 30%, a total sample of 2542 participants is needed (508 control, 2034 intervention).

## Data collection

### Primary outcome

#### Depressive symptoms

The primary outcome is change in depressive symptom, operationalised as the difference in total scores of the PHQ-9 between the baseline and 12-month follow-up, mean difference scores between the intervention and control group will be compared. For intervention group participants, additional discharge time-point data will be collected for clinical but not research purpose. The validated Chinese version of PHQ-9 will be used [34]; it is a 9-item instrument that assesses the frequency of occurrence of main depressive symptoms during the past 2 weeks. Items are rated on a 4-point scale ranging from 0 (not at all) to 3 (nearly every day), and total score can range from 0 to 27, with higher scores reflecting more severe depressive symptoms. Scores of 5–9, 10–14, 15–19, and 20 and above represent mild, moderate, moderately severe, and severe depressive symptoms levels, respectively. Changes in symptom severity level will be used as sensitivity analysis.

### Secondary outcomes

Secondary outcomes will be assessed at the baseline and 12-month follow-up for both groups, and at an additional discharge time-point for intervention groups for clinical service. Changes at 12-month follow-up from the baseline will be used for analysis.

#### Self-harm risk

Self-harm risk will be assessed using eight items adapted from the Self-Harm Inventory [42], providing an overall assessment based on the total score and clinical judgement. Trained social workers assess participants’ risk of self-harm (yes or no answers to 10 items) and harm to others (yes or no answers to 4 items). They will provide an overall evaluation of suicidal risk score, which ranges from 0-No to 3-High.

#### Anxiety symptoms

Anxiety will be indicated by the total scores measured using the Generalized Anxiety Disorder 7-item scale (GAD-7) [43], a 7-item scale in which responses to each anxiety symptom are rated on a 4-point Likert scale of its frequency of occurrence from 0 (not at all) to 3 (nearly every day), with total scores ranging from 0 to 21.

#### Loneliness

The UCLA Loneliness Scale (UCLA-3) [44], a 3-item self-report, will be used to measure an individual’s perceived loneliness, and the total scores will be compared between time-points across different groups. Each item is evaluated with scores ranging from 0 (never) to 3 (often), the total score is the sum of all items, and a higher score indicates a higher level of perceived loneliness.

#### Quality of life

The EQ-5D-5L [45] will be used to measure participants’ quality of life. The EQ-5D-5L is a generic preference-based measure of health on five dimensions (5D): mobility, self-care, usual activities, pain/discomfort, and anxiety/depression, each with five levels (5 L) of problems. The traditional Chinese version for Hong Kong developed by EuroQol Group will be used. EQ-5D-5L health states will be converted into a single index utility score using a scoring algorithm based on public preferences [46].

#### Service utilisation

A locally adapted short version of the Client Service Receipt Inventory (CSRI) [47] will be used to collect details of the current type and level of social services and health services received by each participant. The economic evaluation in this study will focus mainly on the direct service utilisation cost, capturing the actual expenditure relating to general and mental health services provided by the public sector. We will calculate annual expenditure by multiplying the mean of the volume of service usage and unit cost of these services obtained from the government [48, 49]. As the time horizon of the study will be one year, costs will be not discounted. All costs will be converted to US dollars using the official exchange rate.

### Exclusion criteria

#### Cognitive functioning

The Hong Kong Montreal Cognitive Assessment 5-Minute Protocol (HK-MoCA 5-Min) [50], a validated and reliable cognitive screen for stroke and transient ischemic attack, will be used to assess participants’ attention, verbal learning and memory, executive functions/language, and orientation. Participants who scored below the 2nd percentile by age and education on HK-MoCA 5-min were excluded from later analyses to avoid potential insight problems.

## Data analysis

### Effectiveness analysis of primary and secondary outcomes measures

We will follow the Transparent Reporting of Evaluations with Nonrandomized Designs (TREND) statement [51] to undertake data analysis. Interim evaluation of the primary outcome between groups will not be performed because of the rolling nature of the recruitment of both groups. The dataset will be stored in a secure server once it passes the double-entry quality control, and the final dataset will be merged after all follow-up assessments are completed. Multiple imputation models for missing values will be conducted to investigate the potential effects of missing data and attrition, to serve as sensitivity analysis. We will generate descriptive statistics of the demographic and clinical variables at the baseline (T0/baseline) assessment. The independent samples *t*-test will be used to compare the means of continuous variables in the intervention and control groups, the chi-square test will be used to compare categorical variables, and Fisher’s exact test will be used for variables with low cell counts (*n* < 5). The independent samples Mann–Whitney U-test will be used for the majority of clinical scales that are ordinal in nature to examine whether the same distributions could be assumed across groups. As observations at two time points are nested within individuals and individuals are nested within clusters (centres), changes in outcome measures at 12-month follow-up from the baseline, including PHQ-9, self-harm risk, GAD-7, and UCLA-3 scores, will be assessed by a three-level linear mixed model. Because the number of clusters is small, degree-of-freedom correction using the Satterthwaite method will be applied to the mixed models to maintain the desired type I error rate [52]. We will use STATA 14.1 [53] for data analysis. For all tests, the statistical significance level will be set at .05.

### Cost-effectiveness analysis

We will follow the National Institute for Health and Care Excellence (NICE) guideline [54] to conduct the cost-effectiveness analysis by calculating the incremental cost-effectiveness ratio (ICER) in terms of quality-adjusted life years (QALYs) in the intervention group as compared to the control group [55]. ICER captures the ratio of difference in costs (the cost of the intervention group minus the cost of the control group) divided by the difference in effects (the effect of the intervention group minus the effect of the control group).

Costs of the intervention itself will be estimated. Intervention costs in JoyAge include costs for social workers, peer supporters, training costs, programme expenses, administration costs, and costs for office space and IT equipment and software. Due to the differences in preventive care (selective and indicated) and treatment, we separately estimate the cost for these two groups. The costs for these two groups are assumed to be varied in the costs for social workers, who are the main service providers, while the remaining cost categories, or called general resources, are assumed to be the same for all the participants. A time survey will be developed to collect the time used among social workers for preventive care and treatment groups. This will allow us to calculate the cost associated with staff time required to provide preventive care and treatment interventions. The cost of general resources will be divided by the number of participants to provide a per-participant cost. In addition to the intervention cost, the total cost calculations will include expenditures of health and social care utilisation collected through CSRI and be based on a societal perspective. We will set the time-horizon at one year, therefore costs will not be discounted. Its uncertainty will be graphically represented in the ICER plot using the bootstrap method. The cost-effectiveness acceptability curves will be plotted. Sensitivity analyses will be undertaken to ascertain the robustness of the findings.

## Dissemination plans

Results of this study will be published in peer-reviewed scientific journals that have open-access options and presented at local, regional, and international conferences in relevant disciplinary fields. Topics suggested for presentation or publication will be circulated in the research team and the person making the suggestion may be considered as the lead author. Substantive contributions to the design, conduct, interpretation, and reporting of this study’s data will be recognised through the granting of authorship. No professional writers will be used. Additional dissemination will be through the JoyAge website, social media platforms (Facebook, Twitter, and YouTube), and traditional media outlets including local newspapers, radio, and TV media.

## Discussion

The JC JoyAge project is the first large-scale study in the world to investigate the effectiveness and cost-effectiveness of a collaborative stepped care and peer support programme for the prevention and treatment of sub-threshold depressive symptoms in community-dwelling older adults.

We hypothesise that the JoyAge model will be more effective and cost-effective compared with the CAU. In the primary outcome, we hypothesise that JoyAge group participants will show greater improvement than CAU. To elaborate on depressive symptoms, we expect the indicated prevention element in the collaborative stepped care model will be more effective than CAU in preventing depression among those who are at risk or have mild depressive symptoms at baseline, and the treatment will be more effective than CAU in treating depressive symptoms among those who have moderate or above sub-threshold depressive symptoms at baseline. In addition, we expect that the ICER in the collaborative stepped care model will be lower than the cost-effectiveness threshold suggested by NICE [54], hence more cost-effective.

This paper details a protocol that aligns services from different units to address the fragmentation of the existing mental health system, provides a range of services to participants based on needs and changes in needs, and expands the community’s caring capacity by engaging peer supporters through productive ageing activities. The evidence produced from this protocol along with future research may help to guide the reform of current mental health services for elderly mental health, and build a sustainable long-term community with higher caring capacity.

## Limitations

First, the study design is non-randomised and non-blinded due to several feasibility considerations. JoyAge is a complex intervention model that introduces a new collaborative workflow between two different service units, i.e., age care and mental health service units, provides standardised training to all service providers at the same time, and promotes mental health to increase public awareness at the community level. Therefore, centre level is not an appropriate unit of randomisation and stepped wedge design is not a viable option, and we choose district as a unit of comparison. In addition, community aged care centres are open to all community older adults age 60 years and over, and provide both organised programmes for members to participate and free space for them to mingle, for example, a corner to watch TV together and a canteen to have meals and chat. Therefore, randomisation at the individual level may result in contamination of the intervention effect. To sum up, a randomisation trial in the JoyAge context will need to consider the manpower, fund, stakeholder coordination, and operational limits at a whole district level, with a total elderly population of 1.27 million [56], which is beyond the scope of this pilot project. We try to reduce the bias by recruiting participants from the same NGOs in different districts and will address the demographic differences in the statistical analyses.

Second, the data analysts will not be blinded. Due to the uneven sample size of the two groups and the unmatched time-points, that intervention group have three time-points, it is difficult for the data analysts to process missing data without knowing the experimental design. Third, the present study evaluates the effectiveness and cost-effectiveness of the JoyAge model as a whole instead of its individual elements, which is a complex intervention programme composed of difficult elements. It is difficult to delineate the effects of individual elements and investigate the mechanism of change with the current study design. Future studies with triple-blinding randomised design are needed.

## Trial status

At the time of submission (November 2020), the trial is in the follow-up stage. Recruitment and baseline data collection began in 2018 and was completed in December 2019.

## Supplementary Information

**Additional file 1.** SPIRIT checklist.

**Additional file 2: Table S2**: All items from the World Health Organization Trial Registration Data Set (SPIRIT item 2b).

## Data Availability

The dataset collected and/or analysed during the current study is available upon request.

## Abbreviations

CAU: Care as usual
CBT: Cognitive behavioural therapy
CONSORT: Consolidated Standards of Reporting Trials
CSRI: Client Service Receipt Inventory
GAD-7: Generalized Anxiety Disorder-7
HA: Hospital Authority
HK-MoCA 5-Min: Hong Kong Montreal Cognitive Assessment 5-Minute Protocol
IAPT: Improving Access to Psychological Therapies
ICER: incremental cost effectiveness ratio
IMPACT: Improving Mood–Promoting Access to Collaborative Treatment
JC JoyAge: Jockey Club Holistic Support Project for Elderly Mental Wellness
MDD: Major depressive disorders
NGO: Non-governmental organisation
NICE: National Institute for Health and Care Excellence
NIH: National Institute of Health
PHQ-9: Patient Health Questionnaire-9
PS: Peer Supporter
PST: Problem-solving Therapy
QALYs: Quality-adjusted life years
TREND: Transparent Reporting of Evaluations with Nonrandomized Designs
UCLA-3: UCLA 3-item Loneliness Scale
