# Supplementary material for: Collaborative community mental health and aged care services with peer support to prevent late-life depression: study protocol for a non-randomised controlled trial
Source: Trials. 2022 Apr 11;23:280. doi: 10.1186/s13063-022-06122-1 (PMC8996671; doi:10.1186/s13063-022-06122-1)
Supplement: Supplementary file 2 — Additional file 2: Table S2: All items from the World Health Organization Trial Registration Data Set (SPIRIT item 2b). [file 13063_2022_6122_MOESM2_ESM.docx]

Supplementary Table 2: All items from the World Health Organization Trial Registration Data Set (SPIRIT item 2b)

| **Data category** | **Information** |
| --- | --- |
| Primary registry and trial identifying number | ClinicalTrials.gov NCT03593889 |
| Date of registration in primary registry | July 20, 2018 |
| Secondary identifying numbers |  |
| Source(s) of monetary or material support | The Hong Kong Jockey Club Charities Trust |
| Primary sponsor | The Hong Kong Jockey Club Charities Trust |
| Secondary sponsor(s) |  |
| Contact for public queries | Wai-wai Kwok, +852 3917 7445, kwokww@hku.hk |
| Contact for scientific queries | Terry Lum, +852 3917 8569, tlum@hku.hk |
| Public title | JC JoyAge: Jockey Club Holistic Support Project for Elderly Mental Wellness |
| Scientific title | Collaborative community mental health and aged care services with peer support to prevent late-life depression: study protocol for a non-randomised controlled trial |
| Countries of recruitment | Hong Kong SAR, China |
| Health condition(s) or problem(s) studied | Depressive symptoms |
| Intervention(s) | Intervention group: Behavioral intervention  Participants in the intervention group will receive a collaborative stepped care programme provided by registered social workers and trained peer supporters from aged care or mental health service units according to risk levels, symptom severity, and intervention response. Home visits or other contact formats will be delivered by trained peer supporters employed by service units to detect and engage hidden cases.   - For *at-risk* group, 4 weeks of *selective prevention* group sessions will be provided at the elderly service level by trained peer supporters with registered social worker supervision on wellness topics tailored to the person’s concern as an entry point, packaged with mental health information, followed by a review. - For *mild* group, 6-8 weeks of *indicated prevention* with psychoeducation or low-intensity psychotherapy would be provided. - For *moderate* group, 6-8 weeks of *high-intensity* cognitive behavioural therapy (CBT) would be provided.   Control group: Treatment as usual  The control group will receive treatment as usual, which will be determined by the responsible social workers from service units. |
| Key inclusion and exclusion criteria | Inclusion Criteria:   - Age 60 years and older - Reside in Kwun Tong, Kwai Chung, Tseung Kwan O, or Sham Shui Po of Hong Kong - Have one or more known risk factor(s) for developing depression; and/or - Have depressive symptoms of mild level or above; and - Able to give informed consent to participate   Exclusion Criteria:   - Known history of autism, intellectual disability, schizophrenia-spectrum disorder, bipolar disorder, Parkinson's disease, or dementia; and - Imminent suicidal risk; and - Difficulty in communication |
| Study type | Interventional Allocation: Non-Randomized  Intervention model: Parallel Assignment  Masking: None (Open Label) Primary purpose: Prevention |
| Date of first enrolment | October 1, 2017 |
| Target sample size | 2,500+ |
| Recruitment status | Recruiting |
| Primary outcome(s) | Change from baseline depression at 12 months [Time Frame: Baseline and 12-month follow-up]   - Depression will be measured by the Patient Health Questionnaire (PHQ-9). The total score will be used, ranging from 0 to 27. Higher scores indicate higher levels of depressive symptoms. |
| Key secondary outcomes | 1. Change from baseline loneliness at 12 months   Loneliness will be measured by the UCLA loneliness scale (UCLA-3). The total score will be used, ranging from 0 to 9. Higher scores indicate greater loneliness.   1. Change from baseline life engagement at 12 months   Life engagement will be assessed using the typical day interview - a semi-structured interview asking clients about their typical day activities.   1. Change from baseline self-harm risk at 12 months   Self-harm risk will be measured by the self-harm risk assessment checklist.   1. Change from baseline anxiety at 12 months   Anxiety will be measured by the Generalised Anxiety Disorder scale (GAD-7). The total score will be used, ranging from 0 to 21. Higher scores indicate higher levels of anxiety symptoms.   1. Change from baseline cognitive function at 12 months   Cognitive function will be measured by the Hong Kong Montreal Cognitive Assessment 5-Minute Protocol (HK-MoCA 5-Min). The total score will be used, ranging from 0 to 30. Higher scores indicate higher levels of cognitive function.   1. Change from baseline social capital at 12 months   Participants will be asked to list out names of people they would turn to when they feel down and need help for trivial things.   1. Change from baseline health-related quality of life at 12 months   Health-related quality of life will be measured by the EuroQoL 5 Dimensions 5 Levels (EQ-5D-5L).   1. Change from baseline service utilisation at 12 months   Service utilisation will be measured by the Client Service Receipt Inventory (CSRI). |
